# A structural basis for Staphylococcal complement subversion: X-ray structure of the complement-binding domain of *Staphylococcus aureus* protein Sbi in complex with ligand C3d

**DOI:** 10.1016/j.molimm.2010.09.017

**Published:** 2011-01

**Authors:** Elizabeth A. Clark, Susan Crennell, Abhishek Upadhyay, Alexey V. Zozulya, Julia D. Mackay, Dmitri I. Svergun, Stefan Bagby, Jean M.H. van den Elsen

**Affiliations:** a University of Bath, Department of Biology and Biochemistry, Claverton Down, Bath BA2 7AY, United Kingdom; b EMBL, Hamburg Outstation, Notkestrasse 85, Hamburg D-22603, Germany

**Keywords:** *Staphylococcus aureus*, Complement evasion, Complement

## Abstract

The structure of the complement-binding domain of *Staphylococcus aureus* protein Sbi (Sbi-IV) in complex with ligand C3d is presented. The 1.7 Å resolution structure reveals the molecular details of the recognition of thioester-containing fragment C3d of the central complement component C3, involving interactions between residues of Sbi-IV helix α2 and the acidic concave surface of C3d. The complex provides a structural basis for the binding preference of Sbi for native C3 over C3b and explains how Sbi-IV inhibits the interaction between C3d and complement receptor 2. A second C3d binding site on Sbi-IV is identified in the crystal structure that is not observed in related *S. aureus* C3 inhibitors Efb-C and Ehp. This binding mode perhaps hints as to how Sbi-IV, as part of Sbi, forms a C3b–Sbi adduct and causes futile consumption of C3, an extraordinary aspect of Sbi function that is not shared by any other known Staphylococcal complement inhibitor.

## 1 Introduction

Many bacterial pathogens have evolved ways to adapt to their host environment and to survive host immune system attack by producing a variety of immuno-modulating factors. Gram-positive human pathogen *Staphylococcus aureus*, for example, is a leading cause of hospital and community acquired infections (Lowy, 1998) and is a master of immune evasion. *S. aureus* has a vast arsenal of intrinsic factors that can regulate both adaptive and innate immune systems in a variety of hosts and in addition has evolved elements that enable the bacterium to hi-jack host immuno-regulators enabling it to persist in the host environment. Sbi, for instance, binds to factor H in a tripartite complex (Haupt et al., 2008) and ClfA binds to and activates the regulatory protease factor I (Hair et al., 2008).

Currently six intrinsic complement modulators secreted by *S. aureus* have been identified and characterized. They include Staphylococcal complement inhibitor (SCIN) (Rooijakkers et al., 2005), which binds to the classical (C4b2a) and alternative (C3bBb) pathway C3 convertases at a bacterial surface, stabilizing them and inhibiting their enzymatic activity (Rooijakkers et al., 2009). The C-terminal fragment of extracellular fibrinogen-binding protein EFb-C and its homologue Ehp bind to the C3d region of C3, the central complement component. Efb and Ehp interact with the C3d fragment in native C3 as well as within activated C3b, thereby inhibiting C3b deposition on target surfaces (Hammel et al., 2007a,b; Lee et al., 2004). Efb and Ehp binding to C3 has been proposed to induce a conformational change in native C3 so that it can no longer participate in the propagation of downstream activation processes in the cascade amplification pathway (Hammel et al., 2007a,b). Staphylococcal superantigen-like protein 7 (SSL7) affects the terminal pathway by binding to C5 and in so doing inhibits the complement-mediated bactericidal activity of human serum (Langley et al., 2005), most likely by preventing C5 cleavage by C5 convertases. Chemotaxis inhibitory protein of *S. aureus* (CHIPS) (de Haas et al., 2004) binds to the C5a receptor presented on phagocytes in a way that prevents signaling via the inflammatory anaphylatoxin C5a. Finally, *S. aureus* binder of immunoglobulin (Sbi), the most recently characterized member of immuno modulators, affects the adaptive immune system by sequestering host IgG through the formation of insoluble complexes (Atkins et al., 2008). In addition to immunoglobulin binding domains I and II, Sbi contains two further domains (Sbi-III and IV) that can bind C3d (in native C3, iC3b and C3dg) and in concert cause futile fluid phase consumption of C3, the most abundant complement component, through activation of the alternative pathway (Burman et al., 2008). The four N-terminal Sbi domains (I–IV; also referred to as Sbi-E) form a very elongated molecule with a diameter of ∼155 Å (Burman et al., 2008), followed by a proline repeat-containing linker and a tyrosine-rich region of unknown architecture. Although the C-terminal region of Sbi lacks an LPXTG cell-wall anchoring sequence it was previously thought to be attached to the staphylococcal cell wall (Zhang et al., 1999). More recently we have found that Sbi is secreted in the surrounding medium (Burman et al., 2008).

Complement subversion by *S. aureus* displays a high level of redundancy, involving complement inhibitors with very similar binding modes. For example, Sbi domain IV possesses significant structural and functional similarities with Efb-C and Ehp. The three molecules display a common three-helix bundle fold (Hammel et al., 2007a,b; Upadhyay et al., 2008a) and share the two most prominent C3d anchoring residues (R131 and N138, in Efb-C; R75 and N82, in Ehp; R231 and N238 in Sbi) despite minimal overall sequence identity. In addition they inhibit the alternative complement pathway and through their interaction with the same residues on the acidic concave surface on C3d they block the binding of C3d to complement receptor 2 (CR2) (Isenman et al., 2010; Ricklin et al., 2008), thereby interfering with the vital link between the adaptive and innate branches of the human immune system. On the other hand, the mechanism through which Sbi (Sbi-E and Sbi-III–IV) interferes with alternative pathway is very different from that of Efb-C or Ehp. In the presence of domain III, Sbi-IV induces futile fluid phase consumption of complement component C3, involving the formation of a covalent adduct with activated C3b (Burman et al., 2008). More recently, Sbi has been implied in the hi-jacking of host complement regulator protein factor H. Alternative pathway regulators factor H (fH) and factor H-like-1 (FHL-1) were shown to bind to Sbi in complex with C3d, forming a tripartite complex (Haupt et al., 2008).

The structure of the complex between Sbi-IV and C3d presented here provides insight into the structural and functional similarities between Sbi-IV and Efb-C/Ehp. It also potentially helps to elucidate the atomic details of an extraordinary aspect of this domain (as part of Sbi-E and Sbi-III–IV) that is not shared by any known staphylococcal complement inhibitor, the formation of a C3b–Sbi adduct and the futile fluid phase consumption of C3.

## 2 Materials and methods

### 2.1 Cloning, expression and purification of Sbi-IV and C3d

DNA coding for Sbi-IV, comprised of amino acids V198-A266 of the Sbi sequence, was amplified using as template the previously described Sbi-III–IV pHIS-Parallel plasmid (Burman et al., 2008). The following oligonucleotide primers were used: Sbi-IV forward primer (BamHI) CGG GAT CC GTT TCA ATT GAA AAA GCA ATC; Sbi-IV reverse primer (HindIII) CCC AAG CTT TCA TTA CGC CAC TTT CTT TTC AGC. Following sequential restriction digestions with BamHI and HindIII, the Sbi-IV fragment was ligated into a pQE30 vector. Sbi-IV expression in *E. coli* BL21(DE3) (Stratagene) was induced with 1 mM IPTG for 3 h at 37 °C. The cells were harvested by centrifugation (6000 × *g* for 10 min at 4 °C) and resuspended in His buffer A (50 mM Tris, 300 mM sodium chloride, 20 mM imidazole, pH 8.0) to give a total volume of 10 ml. The cells were then kept on ice and sonicated (80% amplitude in 0.5 s pulses with 3 s between pulses for a total sonication time of 1 min) using a Branson 450 Digital Sonifier. A further ∼20 ml His buffer A was added and cleared supernatant containing soluble protein was produced by centrifuging at 60,000 × *g* for 30 min at 4 °C. Cleared lysate was loaded into a 50 ml superloop and connected to an AktaPurifier 10 chromatography unit (GE Healthcare). The 6-His tagged protein was purified on a 5 ml His Trap FF column (GE Healthcare) using an imidazole gradient (0–500 mM) with His buffer B (50 mM Tris, 300 mM sodium chloride, 500 mM imidazole, pH 8.0).

C3d, comprised of amino acids 996–1303 of C3, was previously cloned into the pET15b vector to enable purification by ion-exchange chromatography (Nagar et al., 1998). The N-terminal His tag coding sequence was deleted from this vector such that C3d was expressed without the His tag. The plasmid was transformed into *E. coli* BL21(DE3) cells (Stratagene). 1 L secondary cultures were induced with 1 mM IPTG overnight at 18 °C. Cleared lysate was subjected to ion-exchange chromatography on a 5 ml Hi-Trap (SP FF) column (GE Healthcare) using HEPES buffer A (50 mM HEPES pH 5.5) as the loading buffer and eluting with a linear NaCl gradient to 500 mM in the same buffer.

The Sbi-IV–C3d complex was prepared on a 1 ml His trap HP column (GE Healthcare) using the AktaPurifier. Purified Sbi-IV was applied to a 1 ml His Trap HP column (GE Healthcare) equilibrated with His buffer A. The column was then washed with His buffer A and then purified C3d was applied to the column. After a further wash the complex was eluted using an imidazole gradient of 0–500 mM using His buffer B (50 mM Tris, 300 mM sodium chloride, 500 mM imidazole, pH 8.0). Purified protein complex was then buffer exchanged into 50 mM Tris pH 7.0 prior to crystal trials.

### 2.2 Crystallization, data collection and structure analysis

Sbi-IV–C3d complex (in 50 mM Tris pH 7.0 at ∼15 mg/ml) was subjected to a ProPlex screen and a tacsimate screen, using the ‘sitting drop’ vapour diffusion method. The Sbi-IV–C3d complex produced small needle-like crystals in various conditions of the ProPlex screen within 7 days. Crystals grew in the following ProPlex-96 conditions: 100 mM Tris pH 8.0, 20% (w/v) PEG 4000; 200 mM sodium chloride, 100 mM Tris pH 8.0, 20% (w/v) PEG 4000 and in 100 mM sodium HEPES pH 7.0, 1 M sodium citrate. Large crystals suitable for X-ray diffraction analysis were obtained in 100 mM Tris pH 8.0, 200 mM NaCl, 20% PEG 4000, using micro seading (Sead Bead, Hampton Research).

X-ray diffraction data were collected at the Diamond Light Source (Oxfordshire, UK) on an ADSC Q315 3 × 3 CCD detector on station I04 (at wavelength *λ* = 0.9702 Å). A crystal of the Sbi-IV–C3d complex was removed from the crystallization drop using a cryoloop and was placed into cryoprotectant (20% (v/v) glycerol) containing reservoir solution for 1 min. The crystal was then removed from the drop using a micromount and held in a stream of gaseous nitrogen to facilitate freezing of the crystal. 360 images were collected at an oscillation angle of 1°. Data were processed using the HKL2000 package (Otwinowski and Minor, 1997).

Molecular replacement was carried out with Balbes (Long et al., 2008), using the structure of C3d (Protein Data Bank accession code C3D1) as a search model. Automated rebuilding and refinement was carried out by Arp/wArp (Perrakis et al., 1999). Model building was done with Coot (Emsley and Cowtan, 2004) followed by rounds of refinement using Refmac5, part of the ccp4i software (CCP4, 1994).

The final coordinates of the Sbi-IV–C3d complex have been deposited to the Protein Data Bank (PDB accession code 2wy8).

### 2.3 NMR sample preparations and titration experiments

Preparation of uniformly ^15^N-labelled Sbi-IV for NMR titration experiments was carried out as described before (Upadhyay et al., 2008a). Binding of ^15^N-labelled Sbi-IV to unlabelled C3d was followed by recording ^1^H–^15^N HSQC spectra as a function of Sbi-IV:C3d ratio. The NMR titration was performed as previously described (Upadhyay et al., 2008b; Williams et al., 2004). Briefly, two initial NMR samples were prepared in 0.5 ml NMR buffer (5 mM MES, 100 mM sodium chloride, 1 mM EDTA, 1 mM benzamidine and 10% D_2_O pH 5.5). Sample 1 contained 0.6 mM Sbi-IV (1:0 molar ratio of Sbi-IV:C3d). Sample 2 contained 0.6 mM Sbi-IV, 1.2 mM C3d (1:2 molar ratio of Sbi-IV:C3d). The buffer composition of both samples was identical as both samples were extensively exchanged into the same batch of sample buffer. Throughout the titration the concentration of Sbi-IV was maintained at a constant 0.6 mM and the C3d concentration was varied to give a series of Sbi-IV:C3d molar ratios from 1.0:0.0 to 1:2. A ^1^H–^15^N HSQC spectrum was acquired at each titration point with 512 complex ^1^H points and 192 complex ^15^N points with 16 scans per increment and spectral widths of 8000 Hz in ^1^H and 1219 Hz in ^15^N. The initial NMR samples represented the end points of the titration. Intermediate values of Sbi-IV:C3d were obtained by simultaneously taking equal aliquots from both sample 1 and sample 2 and then transferring the aliquots to the other NMR tube (i.e., from tube 1 to tube 2 and vice versa). This procedure was repeated until a series of twelve ^1^H–^15^N HSQC experiments at Sbi-IV:C3d molar ratios between 1:0 and 1:2 was completed.

NMR experiments were performed on a Varian Unity INOVA spectrometer operating at a nominal proton frequency of 600 MHz, using a room temperature triple resonance 5 mm probe equipped with pulse field gradients (PFG) along the *z* axis. All NMR data processing was performed using NMRPipe/NMRDraw (Delaglio et al., 1995). NMR data were analyzed with Analysis (Vranken et al., 2005).

### 2.4 Small-angle X-ray scattering analysis

Synchrotron radiation X-ray scattering data were collected at the X33 beam line of the EMBL, Hamburg Outstation (DORIS III storage ring at DESY). Solutions of Sbi-E, Sbi-III–IV, Sbi-IV, C3d and complexes of the Sbi protein constructs with C3d were measured at protein concentrations of ∼2, ∼5, and ∼10 mg/ml (sample temperature 10 °C), using a MAR345 image plate detector and sample detector distance of 2.7 m and wavelength *λ* = 1.56 Å, covering the momentum transfer range 0.08 < *s* < 0.45 nm^−1^ (*s* = 4π sin(*θ*)/*λ* where 2*θ* is the scattering angle). Complexes of Sbi constructs were prepared in a 1:1 ratio at concentrations mentioned above. Prior to data collection, dynamic light scattering analysis (Nano-S Zetasizer, Malvern) was used to ensure the monodispersity of the protein samples. To check for radiation damage, two successive 2 min exposures taken on the same sample were compared; no radiation effects were observed. The data were processed using standard procedures and extrapolated to zero solute concentration using the program package PRIMUS (Konarev et al., 2003).

The forward scattering *I*(0) and the radius of gyration *R*_g_ were computed from the entire scattering patterns using the indirect transform package GNOM (Svergun, 1992), which also provided the intraparticle distance distribution function *p*(*r*) and the maximum dimension *D*_max_. The molecular mass of the solute was evaluated by comparison of the forward scattering with that from a reference solution of bovine serum albumin (molecular mass 66 kDa). The estimation of excluded volume *V*_ex_ and low resolution *ab initio* models of Sbi-E, Sbi-III–IV, Sbi-IV, C3d and complexes thereof were obtained using the program DAMMIF (Franke, 2009; Svergun, 1999). The program employs simulated annealing to build a compact interconnected configuration of beads inside a sphere with the diameter *D*_max_ that fits experimental data minimizing the discrepancy:

$$\chi^{2}=\frac{1}{N-1}{\sum_{j}\left[\frac{I_{\exp}(s_{j})-cI_{\text{calc}}(s_{j})}{\sigma (s_{j})}\right]}^{2}$$

where *N* is the number of experimental points, *c* is a scaling factor, *I*_exp_(*s*_*j*_) and *I*_calc_(*s*_*j*_) are experimental and calculated intensities, respectively, and *σ*(*s*_*j*_) is the experimental error at the momentum transfer *s*_*j*_. Ten DAMMIF runs were performed to check the stability of the solution, and the results were averaged using the program DAMAVER (Volkov and Svergun, 2003) to yield the most probable models. Rigid body modeling was performed using the program SAXREF (Petoukhov and Svergun, 2005).

## 3 Results

### 3.1 Structure of the Sbi-IV–C3d complex

To gain understanding of the molecular details of the recognition and inhibition of human complement protein C3 by staphylococcal complement modulator Sbi we determined the three-dimensional structure of the complex between C3 binding domain Sbi-IV and the thioester-containing fragment C3d of complement component C3. The crystal structure of the Sbi-IV–C3d complex was determined at a resolution of 1.7 Å with a single molecule of Sbi-IV bound to one molecule of C3d in the asymmetric unit (for data collection statistics see Table 1). This complex was refined to 1.7 Å resolution with *R*_cryst_ and *R*_free_ values of 16.9% and 20.5%, respectively (see Table 2). During initial analysis of the structure it became evident that the interaction between C3d and Sbi-IV involved two separate binding modes including a second symmetry-related Sbi-IV molecule.

**Fig. 1:**
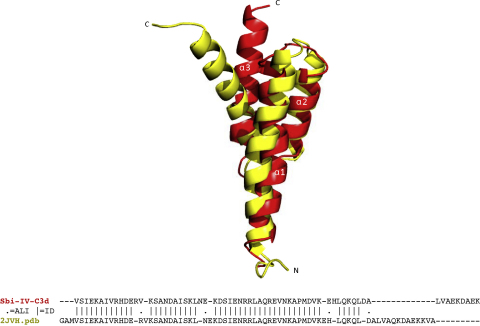
Superposition of complexed and uncomplexed Sbi-IV molecules. Sbi-IV molecules observed in solution (yellow) and in the crystalline complex with C3d (red) are superimposed. A sequence alignment representation of the structural alignment between Sbi-IV in complex with C3d (sequence 1) and the Sbi-IV solution structure (Protein Data Bank accession code 2JVH) (sequence 2) is also shown. (For interpretation of the references to color in this figure legend, the reader is referred to the web version of the article.)

While the structure of C3d in both complexes, when compared with a previous uncomplexed structure [PDB accession code 1C3D (Nagar et al., 1998)], revealed no significant structural changes in its classical α-α6 barrel fold (alignment within 3.5 Å, r.m.s.d. 0.7 Å), notable structural changes were observed between the previously described solution structure of Sbi-IV [PDB accession code 1JVH (Upadhyay et al., 2008a)] and C3d-bound Sbi-IV. This is reflected in the structural alignment of 54 residues in the free and bound state of Sbi-IV revealing a 14-residue gap at the C-terminal end of the molecule (Fig. 1; alignment of residues within 3.5 Å, r.m.s.d. 1.8 Å). In the crystal structure, helix α3 is positioned significantly closer to the α1 and α2 helices, resulting in a more compact three-helix bundle fold. The structural alignment further reveals several smaller structural differences between the solution and X-ray structure, including residues R210 and V211 located within the α1 helix, N222 and E223 (α2–α3 loop), and residues E246 and H247 of the N-terminal part of the α3 helix.

### 3.2 Sbi-IV–C3d interactions

**Fig. 2:**
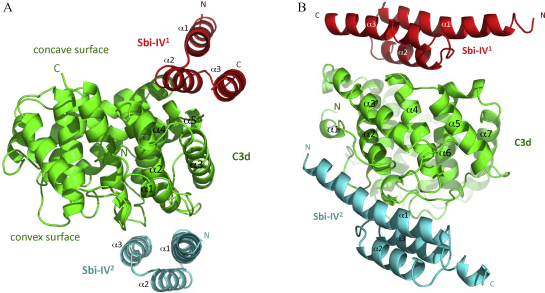
Ribbon representations of the two Sbi-IV–C3d complexes in different C3d side view orientations (A and B). C3d is shown in green, Sbi-IV in complex 1 in red, and Sbi-IV in complex 2 in turquoise. The positions of the concave and convex faces of C3d are indicated. The N- and C-termini of each molecule are indicated and the interacting α helices of C3d and both Sbi-IV molecules are specified. All molecular figures were prepared using MacPyMOL (www.pymol.org). (For interpretation of the references to color in this figure legend, the reader is referred to the web version of the article.)

Similar to complexes of staphylococcal complement inhibitors Efb-C and Ehp with C3d (Hammel et al., 2007a,b), in the first binding mode (complex 1) Sbi-IV binds to the edge of the acidic residue-lined concave surface of dome-shaped C3d (Fig. 2A and B). In the second binding mode (complex 2) Sbi-IV contacts the convex face of C3d containing the thioester, an interaction that has not been observed before with any staphylococcal or other complement inhibitors. Both interfaces show high shape complementarity with comparable buried surface areas (b.s.a.) of 1500 Å^2^ (complex 1) and 1300 Å^2^ (complex 2), accounting for 31% and 26% of the Sbi-IV surface, respectively. These b.s.a. values are comparable to that of the Efb-C–C3d complex [1600 Å^2^; calculated using PISA (Krissinel and Henrick, 2007)]. Below we describe the interactions observed in both complexes in more detail.

#### 3.2.1 Complex 1: Sbi-IV interactions with the concave surface of C3d

**Fig. 3:**
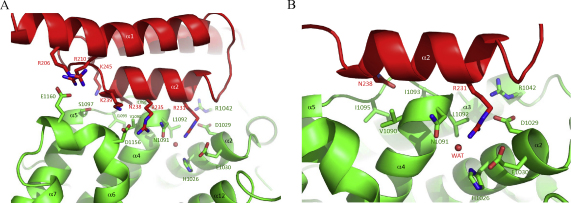
Close-up view of the interacting C3d and Sbi-IV residues in complex 1. A: Ribbon diagrams of C3d (in green) and Sbi-IV (in red) highlighting the main amino acids involved in the interactions between the two molecules at the concave surface of C3d. The interacting residues are represented as stick models (Sbi-IV in red, C3d in green) and are labeled according to full length Sbi and intact pre-pro C3 numbering. B: Detailed view of the interactions involving the two ‘C3d anchoring’ Sbi-IV residues, R231 and N238. (For interpretation of the references to color in this figure legend, the reader is referred to the web version of the article.)

Sbi-IV interacts with the concave surface of C3d mainly through its helix α2 residues (I228, E229, R230, R231, Q234, R235, N238; intact Sbi numbering) with additional contributions from amino acids in helices α1 (R209 and R213) and α3 (K245) (detailed in Fig. 3A). C3d contributions to the interface involve residues from the acidic 30s–40's cluster, connecting helices α2 and α3 (including D36/1029, E37/1030 and R49/1042; C3d numbering/intact pre–pro C3 numbering), loop residues connecting helices α4 and α5 (N98/1091, L99/1092, I100/1093, I102/1095 and S104/1097) and helices α6 and α7 (D163/1156 and E167/1160, the acidic 160's cluster). In the structure of complex 1, Sbi-IV helix α2 residue R231 anchors deeply into a pocket in the acidic 30's–40's cluster in C3d, stabilized by a structural water molecule. On the C-terminal side of helix α2, N238 is involved in an elaborate hydrogen bond network with main-chain atoms of residues in the loop connecting α4 and α5 of C3d, including V97/1090, I100/1093, I102/1095 and S104/1097 (detailed in Fig. 3B). All complex 1 interactions are listed in Table 3.

#### 3.2.2 Complex 2: Sbi-IV interactions with the convex surface of C3d

**Fig. 4:**
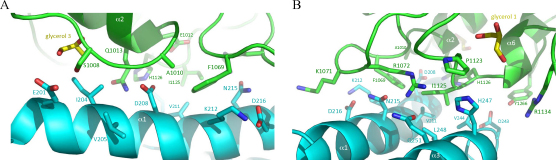
Close-up view of the interactions between Sbi-IV and the C3d thioester region observed in complex 2. A: Ribbon representation of the newly discovered Sbi-IV contact interface at the convex surface of C3d. In this orientation, amino acids at the N-terminus of helix α1 in Sbi-IV (turquoise) are seen interacting with the C3d thioester region residues (in green) B: Detailed view of the interactions made by the C-terminal Sbi-IV residues, including ones from helices α1 and α3. Two glycerol molecules were also found to bind to the thioester region of C3d (shown in yellow; glycerol was used as a cryoprotectant during data collection). (For interpretation of the references to color in this figure legend, the reader is referred to the web version of the article.)

The crystal structure of Sbi-IV in complex with C3d reveals another binding mode, involving a symmetry-related Sbi-IV molecule, which is not observed in the structures of C3d complexed with Efb-C and Ehp. In the Efb-C–C3d and Ehp–C3d complexes, the highly conserved thioester region is obscured by the formation of a dimer of two C3d molecules in the crystal, whereas in complex 2 Sbi-IV helices α1 and α3 form a highly complementary interface with this hydrophobic region (see also Supporting Figure 1). The thioester region interface seen in complex 2 includes residues from loop regions connecting C3d helices α1 and α2, α3 and α4, and α5 and α6. At the core of the interface lie intimate hydrophobic contacts between residues V211, V244 and L248 in Sbi-IV and I1125 of C3d. These interactions are strengthened by van der Waals stacking interactions between K212 (Sbi-IV) and F76/1069 (C3d), as is shown in Fig. 4A and B. These hydrophobic contacts are further stabilized by hydrogen bonding interactions involving Sbi-IV helix α1 residues D208, N215, S219 and D243 and Q251 from helix α3 with C3d thioester residues A17/1010, Q20/1013, K78/1071 R79/1072 and Y273/1266 (see Table 4 for detailed list of interactions). In the C3d construct used in our experiments the thioester-contributing cysteine residue (C17/1010) is substituted by an alanine. In the complex 2 structure Sbi-IV D208 is sandwiched between the two thioester-forming residues (Q20/1013 and C17/1010, here mutated to A17/1010) that are modified in native C3 forming the thiolactone ring that mediates covalent attachment (Fig. 4A).

Results from site-directed mutagenesis in C3d (Isenman et al., 2010) and in Sbi-IV (Burman et al., 2008; Upadhyay et al., 2008a) together with surface plasmon resonance (SPR) and isothermal calorimetry titration (ITC) validate the formation of complex 1. More so, the results from these techniques indicate that complex 1 is the sole species formed under these experimental conditions, suggesting that complex 2 as seen in the crystal structure may not be a physiologically relevant complex but a crystallization artifact. To expand on these observations, we analyzed the complexes of Sbi-E, Sbi-III–IV and Sbi-IV with C3d using two additional structural techniques, small-angle X-ray scattering (SAXS) and NMR chemical shift analysis. The latter technique was chosen also because of its broad affinity range.

### 3.3 SAXS analysis of the complexes of C3d with Sbi-E, Sbi-III–IV and Sbi-IV

**Fig. 5:**
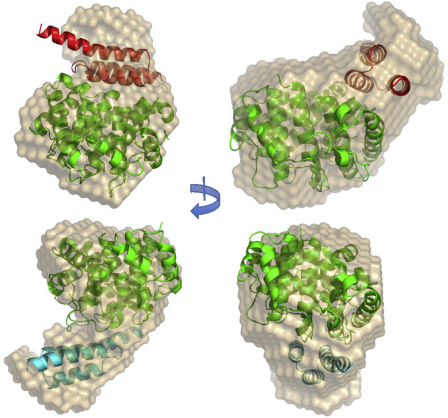
SAXS analysis of the Sbi-IV–C3d complex. Shown are the *ab initio* bead models of the complex determined by DAMMIF and fitted with the structures of complex 1 (top) and complex 2 (bottom). For both complexes two C3d side view orientations are shown.

For further examination of the two C3d binding modes of Sbi-IV observed in the crystal structure, we used small-angle X-ray scattering analysis to study complexes of C3d with Sbi constructs Sbi-E, Sbi-III–IV and Sbi-IV in solution. The results from these analyses are listed in Table 5. In contrast with the dual C3d binding mode seen in the crystal structure, Sbi-IV and all other Sbi constructs form complexes with C3d in a 1:1 stoichiometry. When the atomic coordinates of both complexes 1 and 2 were fitted with the SAXS data, using the program CRYSOL, the resulting *chi* values (complex 1, *χ* = 2.7; complex 2, *χ* = 2.5; Table 6) are inconclusive as to which complex is formed, although there is a slight preference for the model based on complex 2. In concordance with these results, the *ab initio* models and the atomic coordinates of the Sbi-IV–C3d complexes can be superimposed with similarities of 1.5 (complex 1) and 1.4 (complex 2). The *ab initio* bead models for both complexes are shown in Fig. 5. Experimental SAXS data and fitting curves are shown in Supporting Figure 2.

### 3.4 NMR titration analysis of the Sbi-IV residues involved in the interaction with C3d

**Fig. 6:**
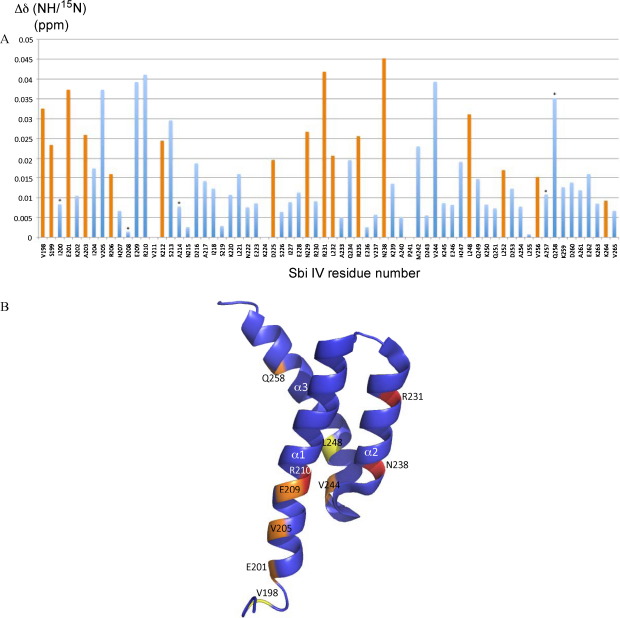
A: C3d-induced chemical shift perturbations in Sbi-IV. Bar chart showing the combined NH proton and ^15^N chemical shift change for all Sbi-IV residues (blue bars). Orange bars indicate peaks that split during titration with C3d, showing the average change across all peaks. Peaks that broaden beyond detection are marked with an asterisk, showing the chemical shift perturbation up to the point of disappearance. B: C3d-induced chemical shift perturbations mapped onto the solution structure of Sbi-IV. Ribbon diagram of the NMR solution structure of Sbi-IV (Upadhyay et al., 2008a) (shown in blue). Positions of residues exhibiting a chemical shift perturbation of >0.04 ppm are highlighted in red, a perturbation of 0.035–0.04 ppm in orange and 0.03–0.035 ppm in yellow. The positions of N- and C-termini and helices α1, α2 and α3 are indicated. (For interpretation of the references to color in this figure legend, the reader is referred to the web version of the article.)

We used NMR titration to gain further understanding of the two interaction modes between Sbi-IV and C3d by examining the complex from the Sbi-IV perspective. ^1^H–^15^N HSQC spectra of ^15^N-labelled Sbi-IV were recorded in the presence of differing concentrations of C3d. The Sbi-IV residues showing the largest C3d-induced chemical shift perturbations are shown in Fig. 6A, and plotted onto the solution structure of Sbi-IV in Fig. 6B. As expected from the interactions observed in complex 1, the most prominent chemical shift changes involve Sbi-IV anchoring residues R231 and N238. Helix α1 residue R210, an additional contact residue in complex 1 also showed a considerable chemical shift perturbation. Notably, peaks arising from other residues in Sbi-IV helices α1 and α3 were also perturbed in the Sbi-IV–C3d titration, albeit to a lesser extent than R231, N238 and R210. Chemical shift perturbations were observed for V244 (located in α2–α3 loop), L248 (α3) and α1 residues E201, V205 and E209. Although these residues can be seen interacting in Sbi-IV–C3d complex 2, they are not detected by either ITC or SPR.

## 4 Discussion

The crystal structure presented here of the complex between complement fragment C3d and domain IV of staphylococcal complement modulation protein Sbi reveals two modes of interaction: (1) charge-driven interactions with the acidic concave surface of C3d, mainly through helix α2 of Sbi-IV (complex 1); (2) hydrophobic interactions with the C3d thioester region, via helices α1 and α3 of a symmetry-related Sbi-IV molecule (complex 2).

### 4.1 Complex 1

The interactions observed in this complex resemble the binding between C3d and staphylococcal complement inhibitors Efb-C (Hammel et al., 2007b) and Ehp (Hammel et al., 2007a). Although there is only 19% sequence identity between Sbi-IV and Efb-C and Ehp, 5 of the 8 identical residues are in the interacting α2 helix of Sbi-IV. These residues include R231 and N238 that have been shown to be essential for the interaction with C3d in all three inhibitors (Hammel et al., 2007a,b; Upadhyay et al., 2008a). In Efb-C and Ehp, these residues (R131/R75 and N138/N82 supported by H130/85) are involved in an intricate network of 7 hydrogen bonds with C3d (Table 7). Sbi-IV–C3d complex 1 is stabilized by 9 hydrogen bonds involving R231 and N238, assisted by R206, R235 and K245. While in Efb-C and Ehp this hydrogen bond network is supported with a single salt-bridge (involving R131/75), in Sbi-IV there are 9 ionic bonds with C3d involving the above-mentioned hydrogen bond network residues.

Although an intricate interaction network consisting of 9 hydrogen bonds and 9 salt-bridges would suggest that Sbi-IV–C3d binding should be higher affinity than C3d with inhibitors Efb-C and Ehp, ITC results prove otherwise. As is shown in Table 7, Efb-C and Ehp bind C3d with a 2 and 3 orders of magnitude higher affinity than Sbi-IV, respectively. This is also reflected by their ability to inhibit the alternative complement pathway by two orders of magnitude. Even though the enthalpy changes seen in the binding of C3d by Efb-C and Ehp point to a more optimal ionic interaction and network of hydrogen bonds, the larger number of Sbi-IV residues contributing to hydrogen and ionic bonds includes charged residues (R231, R206, R235 and K245), containing large aliphatic moieties that may add significantly to the entropically favorable hydrophobic interactions. Also, not all H-bonds and salt-bridges will contribute equally to the complex (Clackson and Wells, 1995) and based on the individual b.s.a. contributions of Sbi-IV residues to the complex we predict that R231 (140 Å^2^), R235 (100 Å^2^) and N238 (80 Å^2^) are the largest contributors, followed by K245 (40 Å^2^) and R206 (30 Å^2^). While in Efb-C the simultaneous loss of the anchoring residues R131 and N138 results in a non-functional Efb-C protein, the individual mutants still form stable 1:1 complexes with no significant structural effects in their complexes with C3d (Haspel et al., 2008). The equivalent residues R231 and N238 in Sbi-IV appear to play a more prominent role in their interactions with C3d as the R231A/N238A double mutant, as well as the individual mutants, completely abolish its C3d binding capacity (Burman et al., 2008; Upadhyay et al., 2008a).

**Fig. 7:**
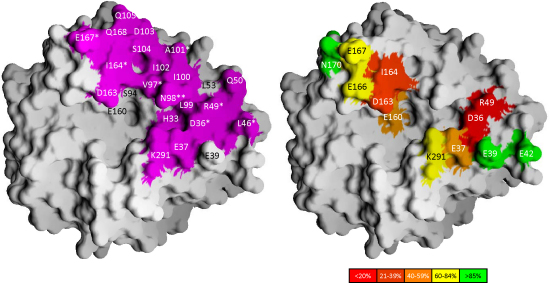
The interaction surface of Sbi-IV on the concave face of C3d. The interaction surface of Sbi-IV on the concave face of C3d as determined in complex 1 (left, colored in magenta) compared with the results from mutational analyses mapped onto the surface rendition of C3d (right, adapted from (Isenman et al., 2010)). Relative binding affinities relative to wild type are color-coded. Residues that contribute the most to the interaction surface in complex 1 are indicated by asterisks (*). (For interpretation of the references to color in this figure legend, the reader is referred to the web version of the article.)

In complex 1, N238 forms intricate hydrogen bond interactions with the C3d main chain, while R231 makes ionic interactions with the side chain of D36/1029. The latter observation is in excellent agreement with recent results from mutational mapping analyses which revealed that the loss of charge mutations in the acidic 30's–40's cluster (D36/1029 and R49/1042) lead to a complete loss of Sbi-IV binding activity (Isenman et al., 2010), while alanine mutations in the other acidic cluster (D163/1156 and I164/1157) caused moderate defects in binding of Sbi-IV. This is also illustrated in Fig. 7 where the results from C3d mutational mapping analyses and the Sbi-IV interaction surface on C3d seen in complex 1 are compared. The C3d amino acids that were tested in the mutational analysis show a substantial overlap with the interaction boundaries observed in complex 1. The importance of C3d residues D36/1029 and R49/1042 determined by the mutational analysis are reflected in this complex by their individual b.s.a. contributions in the complex (40 Å^2^ and 50 Å^2^, respectively). Amino acids D163/1156 and I164/1157, causing moderate binding defects in the mutational analysis, also show to be large surface contributors in complex 1 (60 Å^2^ and 40 Å^2^, respectively). In complex 1 we can identify an additional 5 residues, omitted in the mutational analyses, with considerable b.s.a. contributions. These include 40's cluster residue L46/1039 (50 Å^2^) and 100's cluster residues V97/1090 (50 Å^2^) N98/1091 (100 Å^2^); A101/1094 (50 Å^2^) and I167/1160 (70 Å^2^).

The above-mentioned mutagenesis studies also revealed that the C3d binding site Sbi-IV overlaps with that of CR2. These data were confirmed in the earlier studies in which was shown that Sbi-III–IV (Burman et al., 2008) and also Sbi-IV (Isenman et al., 2010) have the capability to competitively inhibit the binding of C3dg to CR2 (Burman et al., 2008), indicating that complement modulator Sbi also interferes with the link between the innate and adaptive branches of the host immune system. Similar to Efb-C (Burman et al., 2008; Ricklin et al., 2008), the interaction of Sbi-IV with the acidic concave surface of C3d observed in Sbi-IV–C3d complex 1 therefore explains the observed inhibition of the crucial C3d–CR2 interaction by Efb-C and Sbi-IV. However, the discovery of overlapping binding sites for Sbi-IV and CR2 does not fit with the CR2 binding site on C3d observed in a crystal structure of this complex (Szakonyi et al., 2001). As can be seen in Supporting Figure 3, the Sbi-IV molecule in complex 1 (or in complex 2) does not interfere with the interactions between CR2 and C3d in structure described by Szakonyi and co-workers. This controversy has recently been resolved with the finding that zinc ions, at concentrations used in the crystallization conditions, obstruct the binding between C3d and CR2 in the fluid phase, casting doubt on the physiological relevance of the C3d–CR2 interface observed in the crystal structure of this complex (Isenman et al., 2010). It is more likely that the observed interactions are crystallization artifacts, stabilized by the presence of the divalent cations.

The crystal structures of native C3 (Janssen et al., 2005) and C3b (Janssen et al., 2006; Wiesmann et al., 2006) have been very helpful in gaining understanding in the observed Sbi-IV binding modes. Alignment of Sbi-IV–C3d complex 1 with the structures of native C3 (Janssen et al., 2005) and C3b (Janssen et al., 2006; Wiesmann et al., 2006) was used to provide the structural basis for previously observed binding preferences for C3 and its proteolytic cleavage products. While good binding has been reported between Sbi-IV and intact C3, the strongest interactions were observed with iC3b and C3dg and the weakest binding to C3c and C3b (Burman et al., 2008). As was shown for Efb (Hammel et al., 2007b), the superpositioning of the C3d structure in complex 1 with that of the thioester domain (TED) in C3 (Supporting Figure 4) caused no structural interference between Sbi-IV and C3, with opportunities for additional contacts between Sbi-IV helix α3 and C3 macroglobulin domain 2 (MG2). The previously observed low affinity of Sbi-IV for C3b (Burman et al., 2008) can equally be elucidated when complex 1 is superposed onto the structure of C3b. Sbi-IV helix α3 now appears to cause a significant steric clash with C3b macroglobulin domain I (MG1) (Supporting Figure 4, right panel) that would result in disruption of the structure of active C3b. The high affinity of Sbi for C3 cleavage product iC3b on the other hand indicates that in this species the complex 1 Sbi-IV binding site on C3d becomes more accessible.

### 4.2 Complex 2

The presented results from SAXS analysis of the interaction between Sbi and C3d point to the formation of a 1:1 complex between the two proteins, confirming previous SPR and ITC binding experiments with Sbi-IV and C3d (Burman et al., 2008; Upadhyay et al., 2008a). Although it is not possible to distinguish between complex 1 and complex 2 using SAXS, perhaps because of the spherical nature of C3d, results from binding studies with Sbi-IV (Upadhyay et al., 2008a) in combination with mutational mapping studies in C3d (Isenman et al., 2010) identify the interactions observed in complex 1 as the sole functionally relevant species.

NMR chemical shift analysis has a broad affinity range (100 nM to 10 mM) because it can reliably detect even a small percentage of bound ligand (Widmer and Jahnke, 2004). Even low binding affinities in the high millimolar range can be detected, which are beyond the detection limits of ITC or SPR. Our chemical shift analyses of the Sbi-IV–C3d complex clearly show interactions of C3d with both faces of the Sbi-IV molecule that are in concordance with both of the two binding modes observed in the structure. These results indicate that in addition to the high affinity interaction between C3d and Sbi-IV seen in complex 1, another C3d binding site is present on Sbi-IV, represented by complex 2, which is of such low affinity that it can only be detected by NMR and X-ray crystallography. Another feature of the complex 2 binding mode that points to a specific interaction is the significant shape and charge complementarity between the C3d and Sbi-IV molecules, displaying a buried surface area that is on a par with that observed in complex 1, involving 16 residues interacting via 7 hydrogen bonds and covering over a quarter of the available Sbi-IV surface area. For comparison, the b.s.a. observed in complex 2 (1,300 Å^2^) is significantly larger than the crystal packing surface between CR2 and C3d in the structure of the latter complex (800 Å^2^), covering only 8% of the CR2 molecule (Szakonyi et al., 2001).

The crystal structure presented here in combination with the chemical shift NMR analyses indicates that a low affinity short-distance attraction exists between Sbi-IV and C3d, resulting in the interaction seen in complex 2. Perhaps this complex reflects an interaction that is required for the formation of the covalent adduct between Sbi and C3 when incubated with human serum. Even though Sbi-III is needed for activation of C3 it is possible that Sbi-IV forms a significant part of the C3b transacylation target. Interestingly, the N-terminus of the Sbi-IV construct that was used in these studies includes seven residues of the C-terminal sequence of Sbi-III (VSIEKIV, residues 199–205). Although previous NMR solution analysis of Sbi-III and Sbi-IV (Upadhyay et al., 2008a) show that this region is disordered in both molecules, in the Sbi-IV–C3d structure it is fully folded and α-helical, with three residues (E201, I204 and V205) contributing to interactions with the thioester region of C3d (Table 4). These specific interactions are also confirmed in the NMR chemical shift analysis. We are currently further investigating the molecular details of the Sbi-III–IV–C3b adduct.

## 5 Conclusions

We have presented the structure of a domain of staphylococcal complement subversion protein Sbi in complex with a component of the complement system, the first line in the defense against bacterial infections. Similar to *S. aureus* complement inhibitors Efb-C and Ehp, Sbi-IV binds to the thioester-containing domain of native C3. While this C3 binding mode in Efb and Ehp blocks the generation of activated C3b and the propagation of the alternative pathway through its binding to any C3b that does form, the interaction between Sbi-IV and C3, in the presence of domain III, results in the formation of a covalent Sbi-III–IV–C3 adduct followed by futile fluid phase consumption of C3. A second C3d binding site on Sbi-IV, not observed with Efb or Ehp, perhaps hints as to how and where on Sbi this transacylation may occur.

These results not only shed light on the mechanism by which Sbi modulates complement during infection, they also raise the possibility of designing Sbi-based therapeutics for the treatment of complement-mediated diseases. The use of C3 inhibitors in therapy is restricted by the high levels of complement component C3 in plasma, demanding high concentrations of high affinity inhibitors. The use of an inhibitor such as Sbi, which induces C3 consumption, therefore offers a promising alternative for the design of therapeutics involving efficient reduction of C3 levels.

## Figures and Tables

**Table 1:** Data collection statistics.

Unit cell dimensions	53.4 Å × 81.0 Å × 87.7 Å, *α* = *β* = *γ* = 90°
Space group	P2_1_2_1_2_1_
Molecules/asymmetric unit	2
Wavelength	0.9702 Å
Resolution limits	1.7 Å
Temperature	100 K
Total reflections	1369401
Unique reflections (1.70–1.76 Å)	41186 (3710)
*I*/*σI* (1.70–1.76 Å)	30.4 (3.3)
Completeness (1.70–1.76 Å)	96.0% (87.6%)

**Table 2:** Refinement statistics.

	Sbi-IV:C3d complex
Resolution (Å)	45–1.7
*R*	0.1688
*R*_free_	0.2047
Atoms (no.)	3280
Residues (no.)	357
Ordered water molecules (no.)	357
R.m.s.d. bonds (Å)	0.011
R.m.s.d. angles (°)	1.208
Average B-factors (Å^2^)	27.10/21.86

**Table 3:** Sbi-IV interactions with the concave surface of C3d (complex 1).

Sbi-IV residue	Sbi-IV helix	C3d contacting residue(s)
R206	α1	E167/1160
R210	α1	E167/1160, E172/1165
I227	α1–α2 loop	D36/1029, L46/1039, R49/1042
E228	α2	K291/1284
R230	α2	R49/1042
R231	α2	H33/1026, D36/1029, E37/1030, N98/1091, L99/1092
Q234	α2	V97/1090, N98/1091, L99/1092, I100/1093
R235	α2	V97/1090, N98/1091, D163/1156
N238	α2	V97/1090, I100/1093, A101/1094
K239	α2–α3 loop	D203/1196, I204/1197
M242	α2–α3 loop	D103/1096, Q105/1098
K245	α3	I102/1095, S104/1097
Q249	α3	A101/1094
V256	α3	A101/1094
D260	α3	Q50/1043
K263	C-terminus	L46/1039

**Table 4:** Sbi-IV interactions with the convex surface of C3d (complex 2).

Sbi-IV residue	Sbi-IV helix	C3d contacting residue(s)
E201	α1	S15/1008
I204	α1	S15/1008, Q20/1013
V205	α1	Q20/1013
H207	α1	H133/1126
D208	α1	A17/1010, Q20/1013, F76/1069
V211	α1	I132/1125, F76/1069
K212	α1	F76/1069
N215	α1	R79/1072, K78/1071
S219	α1	K78/1071
D243	α2–α3	R141/1134, Y273/1266
V244	α2–α3	I132/1125
H247	α3	P130/1123, I132/1125
L248	α3	I132/1125
Q251	α3	R79/1072

**Table 5:** Overall parameters evaluated from SAXS data.

Sample	*R*_g_ (nm)	MM_exp_ (kDa)	MM_calc_ (kDa)	*D*_max_ (nm)	*V*_p_ (nm^3^)	*V*_ex_ (nm^3^)
C3d	2.17	34.4	34.7	6.7	54	53
Sbi-IV	1.65	11.2	10.9	7.5	23	25
Sbi-III–IV	3.15	17.2	16.5	11.5	32	38
Sbi-E	3.87	30.6	30.7	14.2	67	78
Sbi-IV–C3d complex	2.72	44.0	45.6	8.0	71	70
Sbi-III–IV–C3d complex	3.10	52.6	51.2	12.0	77	75
Sbi-E–C3d complex	3.67	67.0	65.4	13.5	102	123

*R*_g_, MM_exp_, *D*_max_, *V*_p_ and *V*_ex_ are the radius of gyration, molecular mass (MM), maximum size, Porod volume and excluded volume derived from experimental SAXS data. MM_calc_ is the MM calculated from primary sequence.

**Table 6:** Summary of SAXS data modeling fits.

Sample	*χ*_crysol_	*χ*_dammif_	*χ*_RB_	Structure/model
C3d	2.97	–	–	Crystal structure
Sbi-IV	1.78	–	–	Crystal structure
Sbi-III–IV	–	1.44	–	*Ab initio*
Sbi-E	–	1.76	–	*Ab initio*
Sbi-IV–C3d complex	2.75 (complex 1) 2.52 (complex 2)	1.88	–	Crystal structure
Sbi-III–IV–C3d complex	–	1.58	4.49 (complex 1) 4.03 (complex 2)	2 rigid body models (based on the 2 binding modes of the Sbi-IV–C3d complex)
Sbi-E and C3d	–	2.55	–	*Ab initio*

**Table 7:** Comparison of C3d binding data from Sbi-IV, Efb-C and Ehp.

	ITC				Inhibition (alternative pathway)	Structure		
	*n*	*K*_d_ (nM)	Δ*H* (J mol^−1^)	Δ*S* (J mol^−1^)	IC_50_ (μM)	Interface b.s.a. (Å) (Krissinel and Henrick, 2007)	H-bonds	Salt-bridges
Sbi-IV (Upadhyay et al., 2008a)	1.15	360	−15867	69.9	10	1535	9	9
Sbi-III–IV (Upadhyay et al., 2008a)	1.04	400	−19171	58.2	0.14	n/a	n/a	n/a
Efb-C (Hammel et al., 2007b)	∼1	2	−35564	48.1	0.41	1658	7	1
Ehp (Hammel et al., 2007a)	0.94	0.18	−36610	62.8	0.12	1613	7	1
