# Supplementary material for: A structural basis for Staphylococcal complement subversion: X-ray structure of the complement-binding domain of Staphylococcus aureus protein Sbi in complex with ligand C3d
Source: Mol Immunol. 2011 Jan;48(4):452–62. doi: 10.1016/j.molimm.2010.09.017 (PMC3025320; doi:10.1016/j.molimm.2010.09.017)
Supplement: Supplementary file 1 [file mmc1.ppt]

## Slide 1
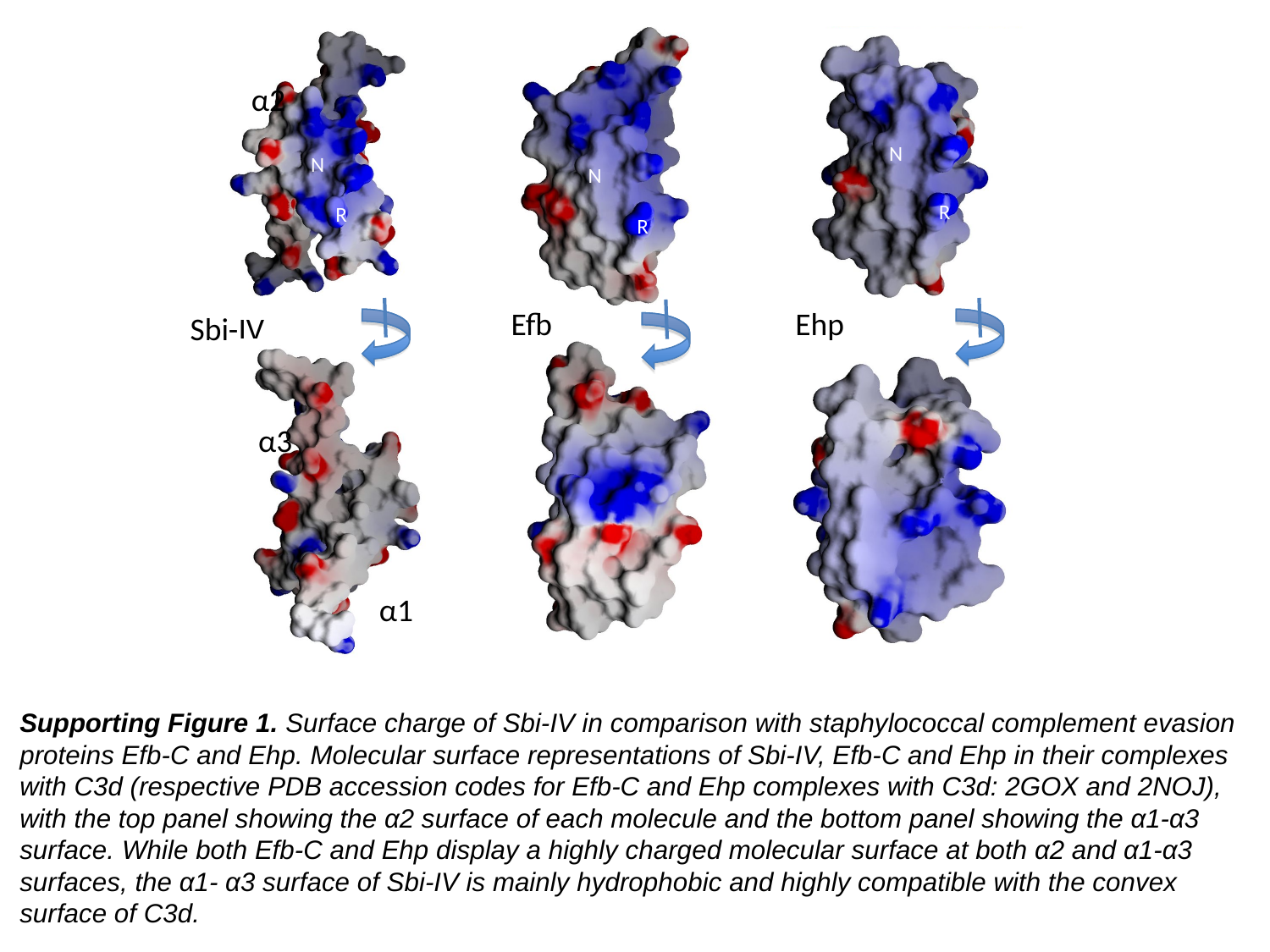

α2
N
N
N
R
R
R
Efb
Ehp
Sbi-IV
α3
α1
Supporting Figure 1. Surface charge of Sbi-IV in comparison with staphylococcal complement evasion proteins Efb-C and Ehp. Molecular surface representations of Sbi-IV, Efb-C and Ehp in their complexes with C3d (respective PDB accession codes for Efb-C and Ehp complexes with C3d: 2GOX and 2NOJ), with the top panel showing the α2 surface of each molecule and the bottom panel showing the α1-α3 surface. While both Efb-C and Ehp display a highly charged molecular surface at both α2 and α1-α3 surfaces, the α1- α3 surface of Sbi-IV is mainly hydrophobic and highly compatible with the convex surface of C3d.

## Slide 2
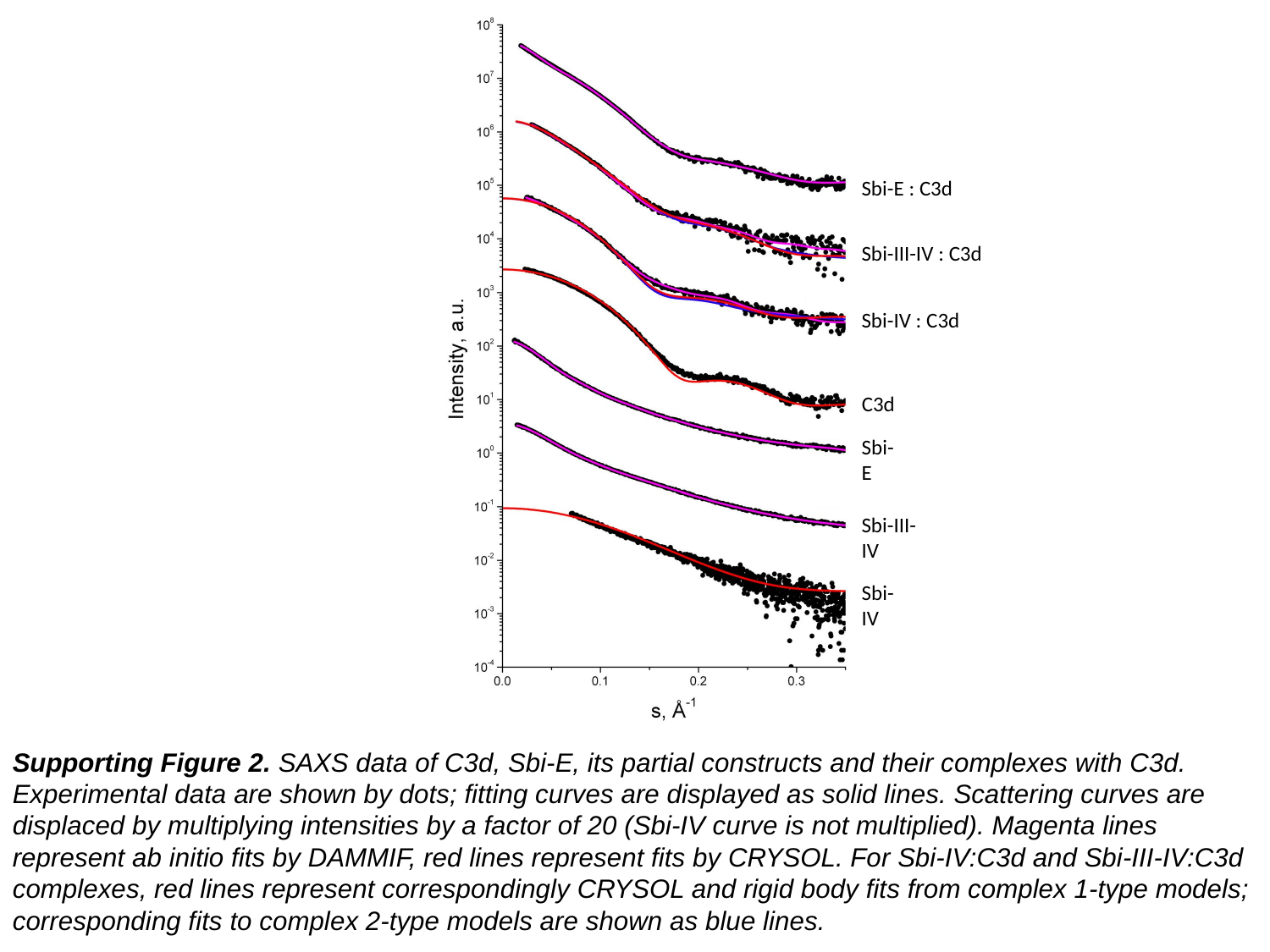

Sbi-E : C3d
Sbi-III-IV : C3d
Sbi-IV : C3d
C3d
Sbi-E
Sbi-III-IV
Sbi-IV
Supporting Figure 2. SAXS data of C3d, Sbi-E, its partial constructs and their complexes with C3d. Experimental data are shown by dots; fitting curves are displayed as solid lines. Scattering curves are displaced by multiplying intensities by a factor of 20 (Sbi-IV curve is not multiplied). Magenta lines represent ab initio fits by DAMMIF, red lines represent fits by CRYSOL. For Sbi-IV:C3d and Sbi-III-IV:C3d complexes, red lines represent correspondingly CRYSOL and rigid body fits from complex 1-type models; corresponding fits to complex 2-type models are shown as blue lines.

## Slide 3
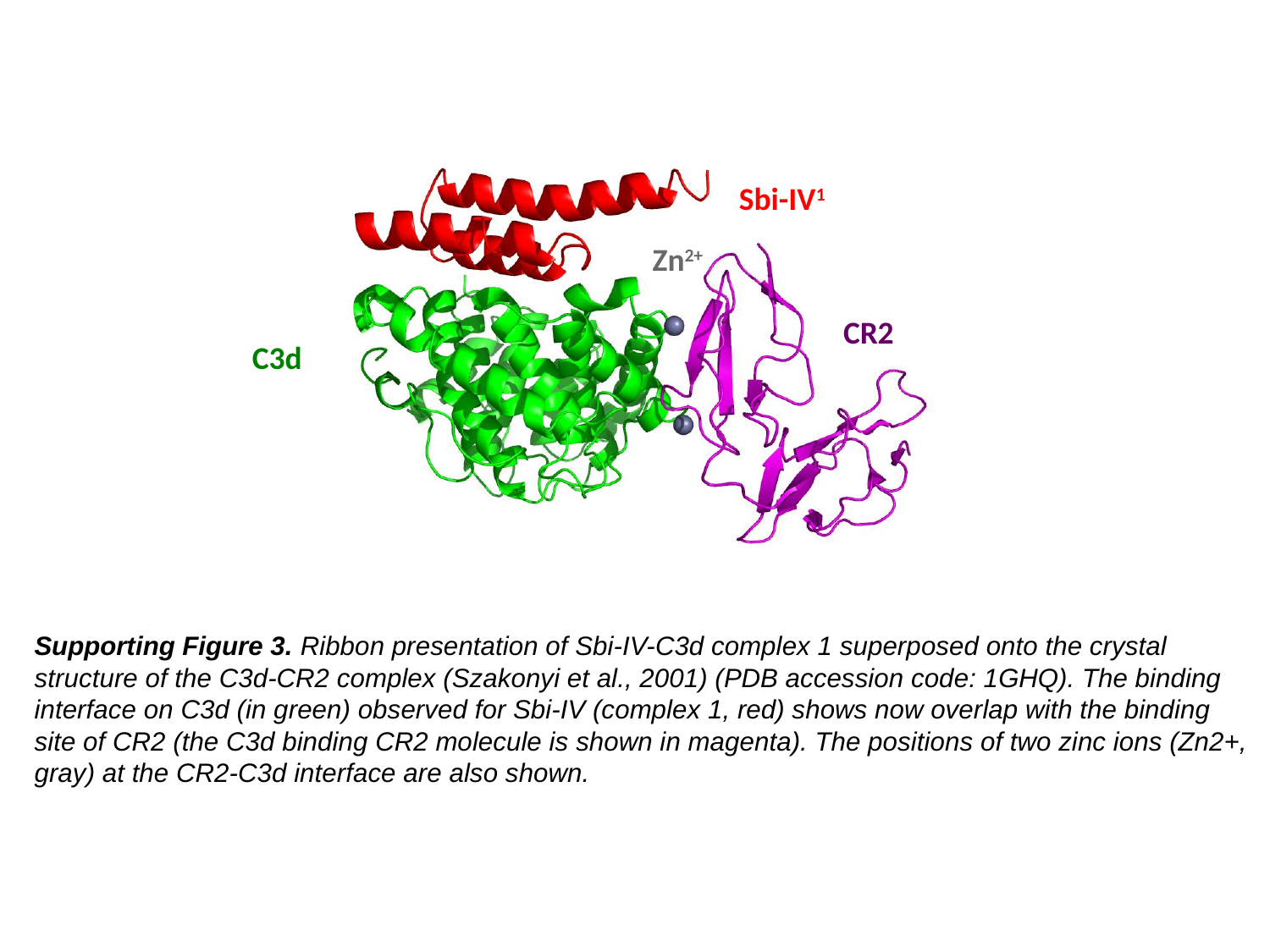

Sbi-IV1
Zn2+
CR2
C3d
Supporting Figure 3. Ribbon presentation of Sbi-IV-C3d complex 1 superposed onto the crystal structure of the C3d-CR2 complex (Szakonyi et al., 2001) (PDB accession code: 1GHQ). The binding interface on C3d (in green) observed for Sbi-IV (complex 1, red) shows now overlap with the binding site of CR2 (the C3d binding CR2 molecule is shown in magenta). The positions of two zinc ions (Zn2+, gray) at the CR2-C3d interface are also shown.

## Slide 4
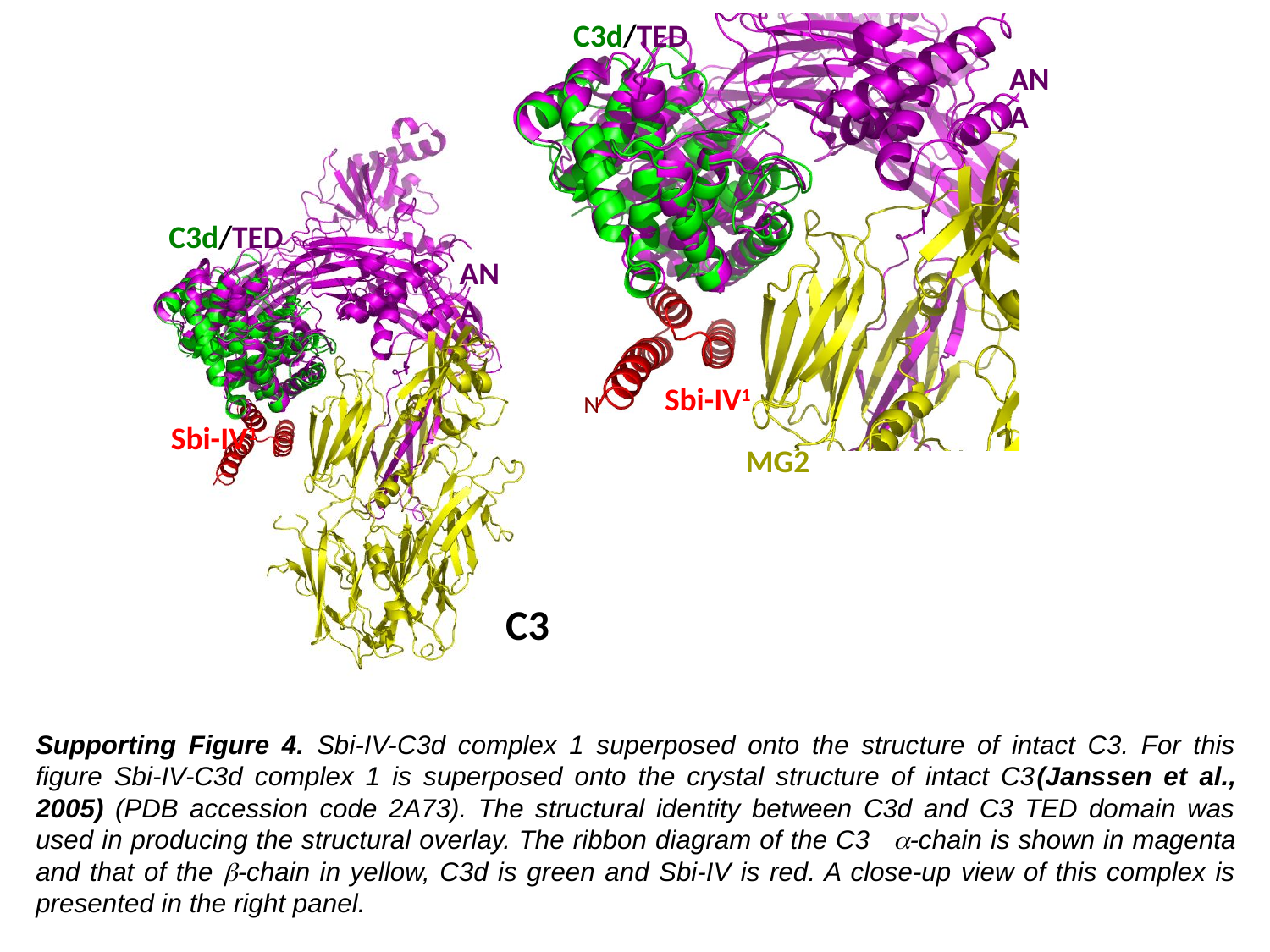

C3d/TED
ANA
C3d/TED
ANA
Sbi-IV1
N
Sbi-IV1
MG2
C3
Supporting Figure 4. Sbi-IV-C3d complex 1 superposed onto the structure of intact C3. For this figure Sbi-IV-C3d complex 1 is superposed onto the crystal structure of intact C3(Janssen et al., 2005) (PDB accession code 2A73). The structural identity between C3d and C3 TED domain was used in producing the structural overlay. The ribbon diagram of the C3 α-chain is shown in magenta and that of the β-chain in yellow, C3d is green and Sbi-IV is red. A close-up view of this complex is presented in the right panel.
